# Views on the well-being of groups of informal caregivers. A cluster analysis using the example of Saxony

**DOI:** 10.1186/s12912-024-02576-7

**Published:** 2024-12-18

**Authors:** Tim Tischendorf, Silke Geithner, Tom Schaal

**Affiliations:** 1 University of Applied Sciences Zwickau, Zwickau, Saxony Germany; 2 University of Applied Sciences for Social Work, Education and Nursing Dresden, Dresden, Saxony Germany

**Keywords:** Informal care, Well-being, Cluster analysis

## Abstract

**Background:**

As a result of demographic change, a further increase in the number of people in need of care in Germany can be expected in the future. Nursing activities performed by family members are a central component of care provision. Providing care for people in need of care is increasingly associated with additional physical and psychological stress for informal caregivers. The aim of this study is to identify and characterize groups of informal caregivers with regard to their well-being.

**Methodology:**

The empirical study was based on a cross-sectional survey on home care in Saxony from 2019, which was intended as exploratory work to provide insights into the care situation in Saxony. The central component is a two-step cluster analysis with exclusively informal caregivers.

**Results:**

The net sample size for the cluster analysis comprised 178 subjects who were involved in caring for relatives. The cluster analysis revealed two groups of caregiving relatives in Saxony, which were differentiated by a different experience of stress and various sociodemographic factors.

**Discussion:**

Informal caregivers in Saxony are not a homogeneous group. Depending on various sociodemographic factors or the care effort and situation, they are confronted with different challenges in caring for relatives, which are directly reflected in their well-being. In order to achieve a targeted reduction in the burden on family caregivers, cooperation and constructive collaboration between political decision-makers, care and health insurers, and the various players in health and care provision is required.

**Supplementary Information:**

The online version contains supplementary material available at 10.1186/s12912-024-02576-7.

## Introduction

Germany is undergoing a demographic transition, characterized by a diminishing youth population and a concurrent rise in older demographics [1]. The consequences of these demographic shifts exhibit regional disparities, with Saxony facing a more pronounced situation compared to the rest of the nation. Although many German states record an average age range of 42 to 45 years for their populace, Saxony’s inhabitants exhibit a higher average age of 46.9 years [2].

One of the reasons for demographic change is the increasing life expectancy of the population as a result of advances in medical care, hygiene, etc.. Based on the mortality tables (2018 / 2020) of the Federal Statistical Office, the average life expectancy for newborns is currently 83.4 years for girls and 78.6 years for boys and has more than doubled since statistical records began in Germany [3]. Increasing life expectancy does not correlate with better health [4]. It increases the probability of the occurrence of multimorbidity, i.e., simultaneous illness from two or more chronic diseases [5]. In view of the demographic development, a further increase in the number of persons with age- and disease-related limitations can be assumed in the future. Expressed in figures, this means for Saxony in 2021 a number of persons in need of care according to SGB XI (Social Code Book XI) of more than 310,000 people, which means an increase of 23.9% compared to 2019 [6].

The Survey of Health Ageing and Retirement in Europe (SHARE) emerges as a particularly significant resource in the current state of scientific research. SHARE is a panel database with microdata on health, socio-economic status, and social networks, covering most of the EU and Israel. It has collected three waves of data (2004, 2006, 2010) and has six more planned until 2024. The data includes information on various aspects of life after 50 years old and is available for free to researchers upon registration. SHARE is harmonized with US and UK studies and serves as a model for aging surveys. Its strength lies in its panel design, multidisciplinary approach, and harmonized cross-national design [7]. The European Quality of Life Survey (EQLS) assesses quality of life in various dimensions since 2003, augmenting traditional economic indicators (GDP, income) with environmental and social aspects. EQLS indicators have influenced decision-making and public debates at EU and national levels. In 2008, EQLS revealed increased care burden on employed family members due to smaller families and more working women, necessitating better support for balancing paid work and care responsibilities in the future [8].

Family caregivers are a central component of care provision in Germany. This group includes family members or other people close to the person in need of care. In 2021, there were 4.96 million people in need of care in Germany, of whom 4.17 million (84%) were cared for at home and 790,000 (16%) in institutions [3]. Of the persons in need of care who were cared for at home, 3.12 million were cared for primarily by relatives, without additional outpatient services [3]. The assumption of care services by relatives not only represents the preferred wish of persons in need of care, but also contributes to the maintenance of the health and social system. However, as a result of the involvement of relatives in caregiving activities, they are increasingly confronted with an additional physical as well as psychological burden, which can promote the exposure of corresponding secondary diseases [9, 10]. Supportive measures for the informal caregivers (IC) enable the maintenance of the well-being as well as the health of the lay caregiver. For this reason, it is particularly important to identify and characterize the groups of ICs in more detail in order to support the ability and willingness of caregiving relatives to take over home care in a goal- and resource-oriented manner.

Despite international studies such as SHARE or EQLS, there is a lack of comprehensive representative survey data specific to the federal state of Saxony in Germany, that provides reliable information on the well-being of ICs. The specific stresses in the various caregiving situations are insufficiently taken into account. The aim of this work was to identify the different views on well-being when taking over caregiving services from the perspective of ICs. The assumption of taking over care services refers here to the informal, non-professional care and support provided by private individuals for relatives in need of care. The question arises as to what extent ICs can be categorized into relatively homogenous groups and characterized in terms of specific stresses and strains and their possible effects on their well-being. On this basis, specific burdens of caring relatives in different care situations can be better understood and highly stressed groups can be characterized more closely.

## Methodology

The present, empirical study was based on a cross-sectional survey on home care in Saxony 2019 and was intended as exploratory work to provide insights into the care situation in Saxony as a population descriptive study. The data analysis was a secondary analysis of a dataset not collected by the author, based on the survey by Schaal et al. 2023. The survey, on which the dataset used is based, was conducted from 1st June to 21st December 2019 as a cross-sectional study and sample survey. The sample was recruited from the residents’ registration offices in the federal state of Saxony. The random selection of the study units was not done directly from the total population, but was collected as a stratified random sample according to the number of inhabitants [11].

Data were collected using a questionnaire with 68 items divided into five categories, which was aimed at both ICs and non-caregiver relatives. The questionnaire consisted partly of standardized measuring instruments and partly of self-developed questions for the explicit topic area. Not all of these 68 questions were relevant to the research question, and they did not refer exclusively to ICs, but also to non-informal caregivers. One standardized instrument relevant to the research question of this article is the Burden Scale for Family Caregivers (BSFC). This served as the basis for a valid assessment of well-being. As a scientific measuring instrument, the BSFC enables the subjective burden of caregiving relatives to be determined on the basis of 10 questions. In order to determine differences in the well-being of the participants, the German version, the Häusliche-Pflege-Skala [Home-Care-Scale] (HPS), was adapted [12]. Within the questionnaire, there was a filter question, allowing differentiation between non-caring and caring relatives. The questionnaire was checked for completeness and comprehensibility in a pre-test and adapted or improved according to the weak points [11].

The questionnaire was made available in online and paper format. In compliance with valid procurement and data protection regulations, invitation cards for the survey were sent by mail to individuals from the cleaned reporting data set as part of commissioned data processing. These contained the link to the survey as well as information on data protection, anonymity and voluntariness, whereby the respondents had to agree in writing. In addition, a hotline was set up for people who could not participate in the survey online. In order to reach these persons nevertheless, the questionnaire was sent by post with a stamped return envelope after a telephone request (*n* = 599) [11]. Further information on the structure of the study, representativeness and other aspects can be viewed and downloaded in a previously published paper together with the original questionnaire and data set [13].

The data analysis was carried out with the statistical software SPSS 29 [11]. The target group for the cluster analysis was defined as people aged 45 years or older who have cared for a relative, friend or neighbor in their own home on an approximately weekly basis. A selection of variables was made for the cluster analysis (Table 1), which were eligible for the procedure and the pursuit of the objective and research question. In the first step, the three reduction criteria for the selection of variables included the inclusion of only ICs on the basis of the filter question in the questionnaire. Subsequently, the selection of variables was based on the work on highly stressed groups of caring relatives - results of a cluster analysis by Bohnet-Joschko [14]. In order to establish comparability of the results, those variables were left in the cluster model which were identical or had a high intersection compared to the variable selection of Bohnet-Joschko. In the final step, variables were selected and excluded from further analysis if they contained at least 25% missing values. In sum, the final model for cluster analysis contained nine variables, with a total of 21 inputs (Table 1).

**Table 1:** Overview of the cluster model and corrected scale levels

Category of questionnaire	Question	Variable
Sociodemographic background	51	own gender
	52	own year of birth
	55	own family status
Statements of informal carers	7	relationship to the person in need of care
	11	areas of restriction of the person in need of care
	12	duration of support for the person in need of care
	14	weekly amount of care required
General statements on the topic of care	49	own financial situation
	50	own well-being

Note. The original questionnaire can be found in the supplementary materials. The values of the individual variables are shown in Table 2, column 1

Two-step cluster analysis was used as the cluster analytical method. Cluster analysis methods are used to group research objects into clusters based on characteristics such as gender, salary level and age, ensuring homogeneity within clusters and differentiation between clusters [15]. The two-step clustering procedure involves preclustering cases to reduce computational complexity, followed by hierarchical clustering to determine the optimal number of clusters using Schwarz’s Bayesian Information Criterion [16]. The noise processing for cluster analysis was set at 25% [16]. Since the cluster analysis was performed using both categorical and continuous variables, the log-likelihood measure was used. The maximum number of clusters was set at 15. Cluster differences for the variables of the well-being metric were tested for significance in each case. For this, we first had the skewness and kurtosis output in SPSS and then performed the Shapiro-Wilk test [15]. The significance level for the multivariate analyses was set at *p* < .05.

## Results

**Fig. 1:**
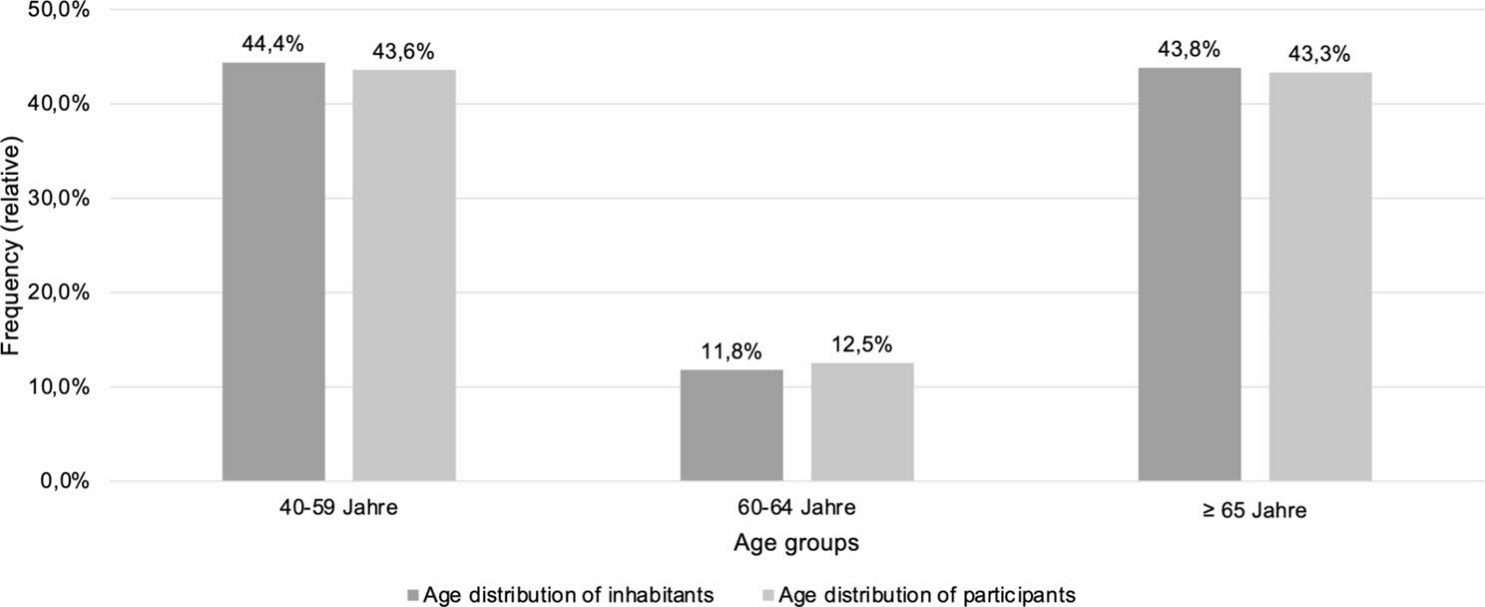
Relative frequencies of participants divided according to age groups compared with the number of inhabitants living in Saxony according to age groups (own representation)

All persons in the identified sample (*N* = 24,018) were invited to participate in the survey. A total of 1,700 valid questionnaires (online and paper versions) were available for evaluation after data cleaning. The net response rate was 7.08% [17]. 1,297 questionnaires (76.3%) were completed online, and 403 paper questionnaires (23.7%) were returned. To assess the representativeness of the sample, the number of inhabitants in Saxony according to three age groups on December 31, 2019 was used as a reference (*n* = 2,460,993) [18]. The sample was representative according to the number of inhabitants living in Saxony by age group and in this respect allows conclusions to be drawn about the population living in Saxony [17] (Fig. 1).

Of the total of 310 persons who had indicated that they had cared for a relative, friend or neighbor in their home or in their own home on an approximately weekly basis in the past twelve months, 132 cases were excluded from the further investigation process because they had not answered at least one of the questions relevant to the model. The net sample size for the cluster analysis thus comprised 178 subjects.

**Table 2:** Overview of clustering characteristics

Characteristics	Cluster		Total
	**1** (42,7%)	**2** (57,3%)	
**Gender**			
female	59,2%	67,6%	64,0%
male	40,8%	30,4%	34,8%
diverse	0,0%	2,0%	1,1%
**Age**			
18 to 25 years	0,0%	0,0%	0,0%
26 to 35 years	0,0%	0,0%	0,0%
36 to 45 years	5,3%	6,9%	6,2%
46 to 55 years	20,8%	30,4%	26,4%
56 to 65 years	34,1%	44,1%	40,0%
over 65 years	39,4%	19,0%	27,7%
**Family status**			
single	1,3%	7,8%	5,1%
married / living in partnership	86,6%	75,5%	80,3%
divorced / separated	2,6%	10,8%	7,3%
widowed	9,2%	5,9%	7,3%
**Nature of the relationship**			
Married partner / life partner	13,2%	18,6%	16,3%
Mother (in law) / father (in law)	60,5%	55,9%	57,9%
(In-law / godfather / foster) child	6,6%	8,8%	7,9%
Other relative (e.g. uncle / aunt, sister / brother, grandchild)	6,6%	4,9%	5,6%
Friend	1,3%	3,9%	2,8%
Neighbour	3,9%	1,0%	2,2%
Other	7,9%	6,9%	7,3%
**Areas of limitation of the person in need of care**			
physical	51,3%	45,1%	47,8%
cognitive / psychological	7,9%	7,8%	7,9%
both	40,8%	47,1%	44,4%
do not know	-	-	-
**Duration**			
under 1 year	21,1%	13,7%	16,9%
1 to under 3 years	31,6%	22,5%	26,4%
3 to under 6 years	30,3%	30,4%	30,3%
6 to under 9 years	7,9%	11,8%	10,1%
9 years and more	9,2%	21,6%	16,3%
There is no care level.	-	-	-
I do not know	-	-	-
**Weekly maintenance effort**			
under 5 h	22,4%	11,8%	16,3%
5 to under 10 h	44,7%	29,4%	36,0%
10 to under 20 h	18,4%	22,5%	20,8%
20 to under 30 h	9,2%	15,7%	12,9%
30 to under 40 h	2,6%	8,8%	6,2%
40 h and more	2,6%	11,8%	7,9%
**Financial situation**			
It’s not enough at all.	1,3%	2,0%	1,7%
I just about manage to get by.	6,6%	27,5%	18,5%
On the whole, I manage.	32,9%	58,8%	47,8%
I am well provided for and can afford a lot.	52,6%	11,8%	29,2%
I don’t have to restrict myself in any way.	6,6%	0,0%	2,8%
**Own well-being in the last four weeks - median figure**			
I have enough time for my own interests and needs.	3	2	3
I often feel physically exhausted.	2	3	3
Every now and then I have the desire to “break out” of my situation.	2	3	2
I can be happy from the heart.	4	3	3
I sometimes no longer really feel like myself.	1	3	2
My standard of living has decreased in recent years.	1	2,5	2
I sometimes feel taken advantage of by people I support.	1	2	1
My current tasks take a lot of my own energy.	2	3	3
I feel “torn” between the different demands of my environment (e.g. work, family, care).	2	3	3
I have the feeling that I have everything “under control”.	3	2	3
Because of my current tasks, my relationship with family members, relatives, friends and acquaintances suffers.	1	3	2
The fate of sick people around me makes me sad.	3	3	3
I get recognition / gratitude through my achievements.	3	3	3

Note. 1 – not true; 2 – little true; 3 – mostly true; 4 – true exactly

The proportion of women was 64.0% (Table 2). The age of the respondents varied between 39 and 84 years (x̄ = 64.02 years (SD ± 0.618)). 80.3% lived in a partnership and 5.1% were single. The remaining 14.6 per cent were either divorced, separated or widowed. Regarding the relationship with the person in need of assistance, the majority of respondents (57.9%) reported caring for their mother (in-law) or father (in-law). 56.7% said they had been assisting the person in need of care for one to six years. In this context, 36.0% indicated a weekly care effort of five to less than ten hours and another 20.8% indicated an effort of ten to less than 20 h. With regard to the assessment of their own financial situation, 47.8% rated it as “I can manage on the whole” and 29.2% as “I am well provided for and can afford quite a bit.”

Information such as “[I] don’t know” or, in the case of the duration category, “There is no care level” were considered missing values in SPSS and excluded from further cluster processing.

## Groups of caregivers

Cluster analysis revealed two groups that reflect the caregiving relatives’ perception of their own well-being in Saxony and its correlation with other factors, as presented in Table 2. The silhouette coefficient, for assessing cluster homogeneity, was in the middle range with a value of 0.3. No normal distribution could be found for the variables of well-being (*p* < .001). Due to the skewed distribution of values and the increased number of outliers, the median was used for the evaluation of the variables of well-being. So-called outliers can affect the average result. In contrast to the mean, these leave the median unaffected [11].

### Cluster 1: Low assistance, good financial situation, high well-being

Cluster 1 was assigned 42.7% (*n* = 76) of ICs (Table 2). The mean age of this group was 62.12 years (SD ± 1.076). The percentage of age, over 65 years old in this group was the highest (39.4%) and also included the highest percentage of men (40.8%). It is also characteristic that 86.6% of the ICs in this group were married or living in a partnership.

**Fig. 2:**
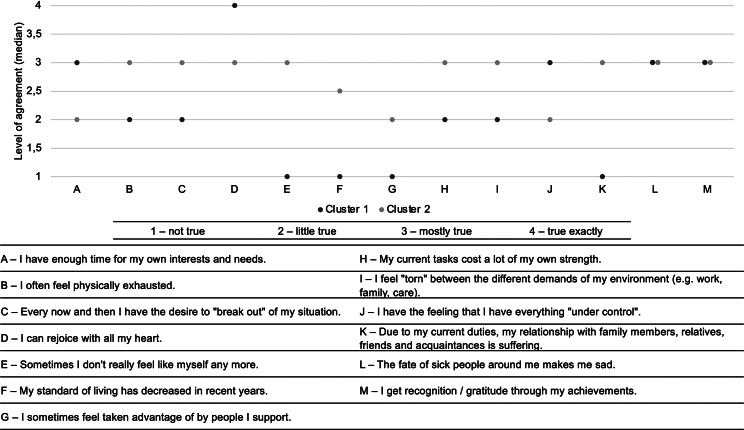
Respondents’ assessment of their personal experience of stress or well-being (*n* = 178)

The cluster is characterized by low participation of family caregivers in support activities. The majority (67.1%) of respondents devoted less than ten hours per week to family caregiving. About half of the respondents (52.7%) had been involved in caregiving for less than three years. With regard to the assessment of their own financial situation, the cluster had the highest percentages for the statements “I am well provided for and can afford quite a bit” (52.6%) and “I do not have to limit myself in any way” (6.6%). With regard to the experience of stress or well-being, the respondents showed a low level of stress (Fig. 2). In all three questions, which according to the BSFC are formulated in terms of a positive sense of well-being, the highest levels of agreement were found in this group. These include “I have enough time for my own interests and needs” (x̃ = 3 (mostly true)), “I can be happy from the bottom of my heart” (x̃ = 4 (true exactly)) and “I feel I have everything under control” (x̃ = 3 (mostly true)).

At 51.3%, the majority of persons in need of assistance in this group had disabilities in the physical sphere. Relatives in need of assistance mostly included mother (in-law) or father (in-law) (60.5%) as well as other relatives or other close persons (7.9%).

### Cluster 2: Intensive assistance, solid to weak financial situation, low well-being

Cluster 2 included 57.3% (*n* = 102) of family caregivers (Table 2). The group included the highest proportion of women (67.6%). The mean age in this cluster was 58.90 years (SD ± 0.884). Again, the majority (75.5%) of respondents reported living in a partnership or being married. Compared to cluster 1, the proportion of single (7.8%) or separated or divorced persons (10.8%) is significantly higher.

Cluster 2 is characterized by a high level of participation in support activities by ICs. Of the participants, 42.2% indicated a duration of three to less than nine years in relation to the question of how long they have been helping the person being cared for. Another 21.6% indicated a duration of nine years or more. With regard to the weekly care effort expended in the process, compared to cluster 1, the respondents in this group consistently showed the highest values from ten hours per week. Here, 11.8% of respondents stated that they invested 40 h or more per week in support activities for the person in need of assistance.

Considering their financial situation, the majority of respondents stated “By and large, I get by” (58.8%). A further 27.5% answered with “I just about get by”. With regard to the assessment of the personal experience of stress or well-being in the past four weeks, the respondents showed an increased level of stress (Fig. 2). The respondents belonging to cluster 2 always showed the highest agreement values for items that are formulated in terms of a high stress experience.

Only the two statements “The situation of sick people in my environment makes me sad” and “My achievements give me recognition / gratitude” did not differ in the two clusters. Both groups indicated a median agreement level of “mostly true”.

Also in cluster 2, the majority (55.9%) of respondents supported the mother (in-law) or father (in-law). When asked in which areas the person to be supported had limitations, 45.1% indicated physical limitations and another 47.1% indicated both physical and cognitive / mental impairments.

Health insurance funds and local authorities can use these findings to design effective support services for highly stressed groups of family caregivers, among others, in a target- and resource-oriented manner.

## Discussion

The aim of this study was to identify and characterize the different views on well-being when taking over nursing services from the perspective of family caregivers. Based on the results of the cluster analysis, two groups emerged that reflect the perception of the caregiving relatives’ own well-being in Saxony and its correlation with other factors. The increasing duration and time spent on caring for relatives and the negative trend in the financial situation of ICs are accompanied by an increased experience of stress and a rather low sense of well-being. While one cluster exhibited low informal involvement in cases of strong financial security among relatives, the opposite was observed in another cluster. This finding demonstrates the consistency of this national survey in relation to international results from SHARE. Canta et al. also concluded that wage ratios are negatively associated with the extent of informal caregiving [19]. Based on the findings of the EQLS, recommendations were made for policymakers and practitioners across Europe to enhance support for informal caregivers. Such improvements can be achieved through training, respite care, counseling, technical aids, and financial assistance [20]. In the German context, the equalization of pension entitlements for caregivers working reduced hours to care for relatives or provide neighborhood assistance is noteworthy. This provision, currently unique to Saxony, addresses the skilled nursing staff shortage and family structure disruption caused by work-related relocations, especially in rural areas. Neighborhood assistance constitutes voluntary civic engagement and not a commercial service, requiring completion of a five-session, 90-minute basic “neighborhood assistance” course financed by care insurance [21]. Despite existing support offerings, demand-side uptake remains incomplete.

In line with the publication by Oltmanns et al. 2016, a positive assessment of one’s own financial situation can have a positive association with subjective well-being [22]. Combining caregiving with a career is often associated with conflicts and difficulties. Concern about their own financial situation can be an additional burden for working ICs, especially if they work full-time [14]. According to Bohnet-Joschko’s findings, although employment can potentially mitigate the negative effects of caregiving and should be maintained if possible, this is controversial because trying to balance work and caregiving often leads to additional stress and strain for the caregiving relatives. Furthermore, in order to provide more comprehensive care for the relative, the number of hours worked is often reduced due to the care provided [23]. It is therefore recommended that working relatives continue to be supported in organizing care and that relief services be developed. It should be noted that the model used does not directly take into account gainful employment itself. The financial burdens addressed here relate to income and financial prosperity, which are closely linked to gainful employment but do not replace it.

Gratitude and empathy are important in informal care and in caregiving in general. There is consistent evidence in the literature of a positive relationship between self-compassion as a personal resource and subjective well-being [24]. While our analysis shows that cluster 2 has lower well-being, the median scores for factors such as sadness about the health of relatives and gratitude received do not differ substantially between the groups. This suggests that other factors, beyond individual levels of gratitude and sadness, may play a crucial role in influencing the differences in well-being between the clusters. Nevertheless, high levels of recognition and gratitude that ICs receive for their services from those they care for generally contribute to greater resilience and may support higher well-being [24].

As the person in need of care becomes more restricted, the caregiving activities become more complex, more extensive and require more time. As a result, the emotional and psychological burden on the family caregiver increases in addition to the physical burden, and they more often feel overwhelmed (cluster 2) [9]. The described physical and psychological burden can be less and less coped with as the age of the family caregiver increases. With retirement age, the proportion of those who support others decreases [23]. However, at older ages (80 years and older), the proportion of caregiving tasks to support provided increases. If people of this age provide help and support to others, it is largely in the form of caregiving activities [13]. The results of the present study reflect this, among other things. While the respondents in cluster 1, who were less involved in caring for their relative, had more than twice the proportion of people over 65, the proportions in the age groups below this in cluster 2 were always the highest.

In accordance with the Bohnet-Joschko cluster analysis, the research results show that ICs who are heavily involved in the care of the relative tend to experience a high level of stress (cluster 2) [14]. A similar picture emerges with regard to the areas of restriction of the person in need of care. Accordingly, relatives who provide support services such as personal hygiene, nutrition and mobility for persons with physical limitations tend to experience high physical stress. High physical strain can lead to physical health problems among ICs themselves [9]. The potential health effects of home care therefore hide the risk of carers themselves becoming patients. They are also referred to as hidden patients or second victims [3]. In this context, respondents in cluster 1 provided the highest proportion of support in the physical sphere, while respondents in cluster 2 provided the highest proportion of care activities for relatives with physical and mental limitations. However, when considering both groups together, the proportion of care recipients with physical limitations remains largely the same across the clusters, with the main difference being that there is a greater proportion of mental limitations in cluster 2. When caring for relatives with mental limitations, ICs may experience increased stress and emotional pressure due to social isolation and constant availability [14]. Despite these differences, the two clusters remain relatively homogeneous in terms of the areas of limitation. Preventive measures, such as teaching caregiving skills and back- and joint-friendly working practices to ICs at an early stage in the provision of basic care services, can play a crucial role in reducing potential health risks to ICs. The results of the study highlight the physical and emotional strains experienced by carers and underline the importance of such preventive measures. For caregivers who are particularly exposed to emotional and psychological pressures, information material and practical tools can offer the opportunity to have more time for themselves and to engage in compensatory activities [14]. Health insurance funds could use these findings to develop targeted prevention programs that not only address physical health but also provide resources for mental well-being, ultimately supporting a healthier and more sustainable caring environment.

More than half of both clusters selected mother (in-law) or father (in-law) as the relationship to the person cared for. Due to the proximity to the person in need of help, the care situation is often emotionally stressful for the adult children [9]. Self-help groups or networks of ICs with similar fates can provide effective support in this regard, offering mutual exchange and support.

## Limitation

With regard to the data collection date in 2019, it should be noted that this was before the Covid-19 pandemic. In addition, there is the general increase in prices as a result of inflation. Due to these multiple pressures, psychological as well as monetary, cluster solutions may have shifted somewhat. Future research could look more closely at and incorporate such a potential difference based on this foundational work.

In accordance with the objective and research question of this study, a selection of variables was made for the cluster analysis. One selection criterion was to exclude variables from further analysis that contained more than 25% missing values. If questions were left unanswered by the subjects, all of the subject’s answers were omitted for the cluster algorithm and he or she was automatically excluded from the clustering. The 25% criterion was intended to ensure that the sample size for clustering was not disproportionately reduced due to a variable with numerous missing values, which could have caused further bias in the study results. In addition to variable selection, only ICs were selected for cluster analysis. 82.0% of the original sample was thus excluded from further consideration against this background.

The silhouette coefficient in the present study was 0.3, indicating mediocre model quality [25]. A mediocre classification corresponds to a weak indication of cluster structure, according to the study by Kaufman and Rousseeuw, on which the classification in SPSS is based [25]. However, to pursue the goal and the research question, it was crucial to include the variables derived from the literature in the cluster analysis. The data set – characterization of groups of ICs. A cluster analysis was uploaded to a data repository to ensure the greatest possible transparency [26].

Cluster analyses allow for the basic desire of people to group objects, facts, etc. into homogeneous groups in order to bring order into a previously confusing situation. It should be noted here that the various generalizing cluster solutions should, however, only be used in an advisory capacity. A limitation of this study is that, due to the methodology, no causal relationship can be established between informal caring tasks and poorer well-being. Instead, it is merely a matter of associations, whereby certain people, as described in the literature, may have a higher tendency to take on intensive caring tasks and at the same time have a poorer sense of well-being. Information and services should continue to be tailored to the most individual situation possible for family caregivers.

## Conclusion

The findings of this study provide a nuanced understanding of the well-being of ICs in Saxony, highlighting the significant impact of factors such as care intensity, financial security and the nature of care tasks. Cluster analysis revealed two distinct groups of caregivers: one with relatively low caregiving involvement and stable financial situation, and another with higher caregiving intensity, greater financial strain and higher stress levels. These findings are consistent with international research and highlight the complex interplay between caregiving, financial well-being and caregiver strain.

The results emphasise that the well-being of people with intellectual disabilities is closely linked to the nature and extent of caregiving responsibilities. For example, caring for people with mental limitations was associated with higher emotional and psychological distress, while intensive physical care was correlated with increased physical strain. These findings point to an urgent need for targeted interventions, such as training in caregiving skills, ergonomic practices and psychological support, to reduce these burdens.

A central lesson is the importance of early prevention and tailored support to address both physical and emotional distress. Policy makers and health insurers could use these findings to develop targeted prevention programmes and improve support structures, including financial assistance, respite care and access to support networks.

Future research should explore causal mechanisms through qualitative studies to deepen understanding of how specific interventions affect caregiver well-being. In addition, research into the long-term effects of caregiving on physical and mental health could inform sustainable care models and policy decisions.

## Electronic supplementary material

Below is the link to the electronic supplementary material.

Supplementary Material 1

## Data Availability

Tim Tischendorf. (2023). Dataset - Characterization of groups of informal carers. A cluster analysis. [Data set]. Zenodo. 10.5281/zenodo.8255875.
