# Supplementary material for: Views on the well-being of groups of informal caregivers. A cluster analysis using the example of Saxony
Source: BMC Nurs. 2024 Dec 18;23:912. doi: 10.1186/s12912-024-02576-7 (PMC11657592; doi:10.1186/s12912-024-02576-7)
Supplement: Supplementary file 1 — Supplementary Material 1 [file 12912_2024_2576_MOESM1_ESM.pdf]

Willkommen bei der Umfrage zur häuslichen Pflege in Sachsen!

Westsächsische Hochschule Zwickau
University of Applied Sciences

Die Umfrage richtet sich an Menschen mit und ohne Erfahrungen in der Pflege. Auch wenn Sie bisher noch keine Erfahrung in der häuslichen Pflege haben, wollen wir Ihre Meinung wissen, denn jede/r kann plötzlich und unerwartet in die Situation kommen, über Pflegeformen entscheiden zu müssen. Häufig wissen betroffene Personen nicht, welche Unterstützung sie erhalten können, welche Leistungen der Kranken- und Pflegeversicherung es gibt und woher sie diese Informationen erhalten können.
Wir freuen uns daher über Ihre Teilnahme an der Befragung! Ihre Meinung ist für die Verbesserung der Pflege in den eigenen vier Wänden wichtig!

Aus allen Haushalten wurden **Sie zufällig** mit Hilfe der Einwohnermeldeämter **ausgewählt**. Die Fragen im Fragebogen beziehen sich auf Ihre Meinung. Wir möchten Sie deshalb bitten, dass Sie persönlich den Fragebogen ausfüllen. Ihre Angaben werden selbstverständlich anonym und vertraulich behandelt.

Die Beantwortung des Fragebogens nimmt ca. **30 bis 35 Minuten** in Anspruch. Die Teilnahme an der Studie ist freiwillig, es entstehen Ihnen keinerlei Nachteile, wenn Sie die Befragung ablehnen oder abbrechen.

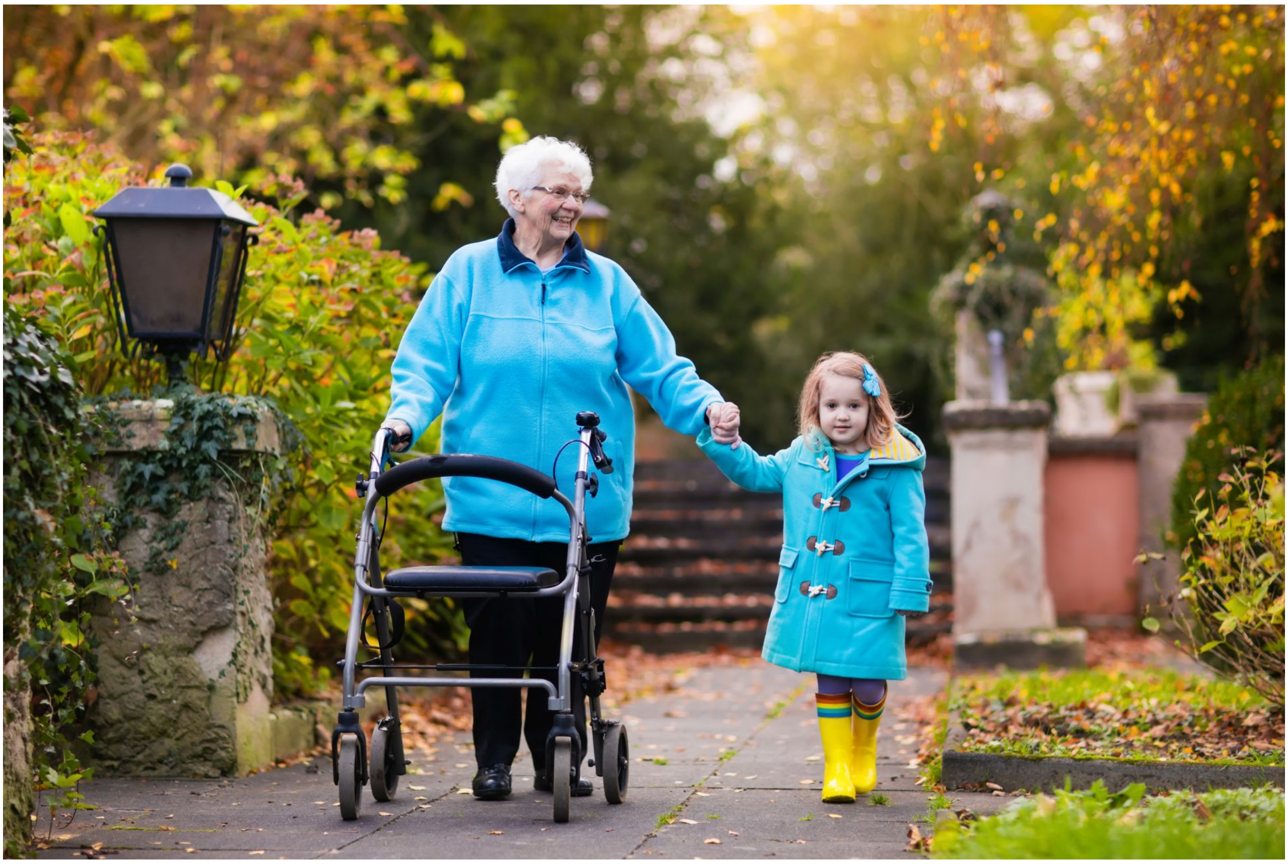

Bearbeitungshinweise: Bitte beantworten Sie jede einzelne Aussage. Entscheiden Sie dabei möglichst spontan. Es gibt keine richtigen oder falschen Antworten, wichtig ist allein Ihre persönliche Einschätzung. Viele der Aussagen werden ähnlich erscheinen, aber sie sind alle notwendig, um Unterschiede in den Bedürfnissen zu zeigen.
Dieser Bogen wird maschinell ausgewertet. Markieren Sie eine Antwort bitte in der folgenden Weise: ○⊗○.
Wenn Sie eine Antwort korrigieren möchten, füllen Sie bitte den falsch markierten Kreis und noch etwas darüber hinaus aus, ungefähr so: ○●⊗.

Ziffern sollen ungefähr so aussehen: 0 1 2 3 4 5 6 7 8 9; Korrekturen so: [illegible]

Bei Fragen zum Projekt, bei Unklarheiten und Problemen beim Ausfüllen des Fragebogens stehen wir Ihnen gern unter der E-Mail: pflege.in.sachsen@fh-zwickau.de oder telefonisch unter **(0375) 536-3421** (Mo 12:00 – 15:00 Uhr und Di 12:00 – 15:00 Uhr) zur Verfügung.

Wir freuen uns daher sehr, wenn Sie uns Ihre wertvollen Erfahrungen in dem Fragebogen mitteilen. Vielen Dank!

Prof. Dr. Tom Schaal, *Projektleiter* und Götz Schneiderat, *Wissenschaftlicher Mitarbeiter*

Datenschutz:
Ihre Angaben können nicht mit Ihrer Person in Verbindung gebracht werden. Die anonymisierten Daten der Befragung werden für wissenschaftliche Zwecke für mindestens zehn Jahre nach Projektende gespeichert. Die von den Meldebehörden zur Verfügung gestellten Adressdaten werden zum Projektende bis zum 31.12.2019 entsprechend DIN 66399 vollständig vernichtet. Die Meldebehörde steht nicht in Verbindung mit dem Inhalt dieses Projektes und hat keinen Einfluss auf etwaige Erinnerungsschreiben. Dritte erhalten keinen Zugang. Den Datenschutzbeauftragten der WHZ, Herrn Dr. Peter Mietke, erreichen Sie unter: (0375) 536-1180 oder peter.mietke@fh-zwickau.de

**Einverständniserklärung**<br>Ich erkläre, dass ich die Informationen zum Datenschutz zur Kenntnis genommen habe und mit der anonymisierten Speicherung und Verarbeitung meiner Angaben, sowie der Veröffentlichung der Ergebnisse in zusammengefasster Form, einverstanden bin. Aufgrund der anonymen Speicherung ist ein nachträglicher Widerruf meiner Angaben nicht möglich.	○ Ja, ich bin einverstanden<br>.

Förderhinweis:

Freistaat Sachsen

Diese Maßnahme wird mitfinanziert mit Steuermitteln auf Grundlage des vom Sächsischen Landtag beschlossenen Haushaltes

MUSTER
Nicht ausfüllen!

Vielen Dank, jetzt können wir mit der Umfrage starten.

1. Wie schätzen Sie die Versorgung von pflegebedürftigen Menschen in Sachsen ganz allgemein ein?

	sehr schlecht										sehr gut
	○	○	○	○	○	○	○	○	○	○	○

Bevor wir Sie weiter zur Pflege in Sachsen befragen, möchten wir Ihre Einstellung zu verschiedenen Themen erfahren. Diese Angaben helfen uns, die Zusammenhänge besser zu verstehen. Außerdem ist für uns interessant, ob und wie sich Menschen, die Angehörige pflegen, von anderen Menschen unterscheiden.

2. Nun möchten wir Sie bitten anzugeben, inwieweit Sie den folgenden Aussagen zur Lebensgestaltung zustimmen!

	stimme gar nicht zu	stimme eher nicht zu	unent-schieden	stimme eher zu	stimme voll zu
Ich verhalte mich stets gesundheitsbewusst.	○	○	○	○	○
Ich habe viele Menschen in meinem Umfeld, an die ich mich immer wenden kann.	○	○	○	○	○
Meine Familie ist mir das Wichtigste.	○	○	○	○	○
In meinem Leben spielt Glaube bzw. Religion eine wichtige Rolle.	○	○	○	○	○
Ich möchte möglichst viel von der Welt sehen.	○	○	○	○	○
Ich habe das Gefühl, dass ich an meinem Wohnort vollständig dazugehöre.	○	○	○	○	○
Es ist mir wichtig, mich für andere Menschen einzusetzen.	○	○	○	○	○
Arbeit stand/steht für mich immer an erster Stelle.	○	○	○	○	○
Ich muss so oft wie möglich raus aus meinen „vier Wänden".	○	○	○	○	○
Ich versuche, in technischen Dingen immer auf der Höhe der Zeit zu sein.	○	○	○	○	○
Ich engagiere mich sehr häufig ehrenamtlich.	○	○	○	○	○
Ich möchte immer etwas Neues lernen.	○	○	○	○	○

3. Jetzt würden wir gern erfahren, wie zufrieden Sie gegenwärtig mit folgenden Aspekten Ihres Lebens sind.

	ganz und gar unzufrieden										ganz und gar zufrieden
Familienleben	○	○	○	○	○	○	○	○	○	○	○
Berufliche Situation bzw. Tageswerk	○	○	○	○	○	○	○	○	○	○	○
Wohnung	○	○	○	○	○	○	○	○	○	○	○
Freundeskreis	○	○	○	○	○	○	○	○	○	○	○
Körperliche Bewegung	○	○	○	○	○	○	○	○	○	○	○
Freizeitgestaltung	○	○	○	○	○	○	○	○	○	○	○
Haushaltseinkommen	○	○	○	○	○	○	○	○	○	○	○
Gesundheit	○	○	○	○	○	○	○	○	○	○	○
Schlaf	○	○	○	○	○	○	○	○	○	○	○
Lebensstandard	○	○	○	○	○	○	○	○	○	○	○

4. Die folgenden Aussagen beziehen sich auf Pflichten, die Menschen möglicherweise in Ihrer Familie haben.

Geben Sie bitte jeweils an, ob Sie voll zustimmen, etwas zustimmen, weder zustimmen noch ablehnen, ob Sie etwas ablehnen oder ganz ablehnen.

	lehne ganz ab	lehne eher ab	weder/ noch	stimme etwas zu	stimme voll und ganz zu
Eltern sollten alles für ihre Kinder tun, selbst auf Kosten des eigenen Wohlergehens.	○	○	○	○	○
Großeltern sollten zur wirtschaftlichen Absicherung ihrer Enkel und deren Familien beitragen.	○	○	○	○	○
Großeltern sollten bei der Betreuung ihrer Enkel helfen, wenn diese noch klein sind.	○	○	○	○	○
Pflegebedürftige Eltern sollten am besten frühzeitig in ein Heim gehen, um ihre Kinder nicht zu belasten.	○	○	○	○	○
Erwachsene Kinder sollten ihre Eltern im hohen Alter bei sich aufnehmen.	○	○	○	○	○
Erwachsene Enkelkinder sollten bei der Betreuung und Pflege ihrer Großeltern mithelfen.	○	○	○	○	○

MUSTER
Nicht ausfüllen!

5. Jetzt geht es um die Unterstützung, die Sie von wichtigen Menschen, also zum Beispiel Ihren Familienmitgliedern, Freunden, Bekannten, Kollegen und Nachbarn erhalten.

Bitte geben Sie an, inwieweit jede der folgenden Aussagen auf Sie zutrifft.

	trifft nicht zu	trifft eher nicht zu	trifft teilweise zu	trifft zu	trifft genau zu
Ich finde ohne Weiteres jemanden, der sich um meine Wohnung kümmert, wenn ich einmal nicht da bin.	○	○	○	○	○
In meinem Umfeld gibt es Menschen, die mich ohne Einschränkung so annehmen wie ich bin.	○	○	○	○	○
Es gibt einen sehr vertrauten Menschen in meinem Leben, mit dessen Hilfe ich immer rechnen kann.	○	○	○	○	○
Bei Bedarf kann ich mir ohne Probleme bei Freunden oder Nachbarn etwas ausleihen.	○	○	○	○	○
Ich kenne mehrere Menschen, mit denen ich gerne etwas unternehme.	○	○	○	○	○
Wenn ich krank bin, kann ich ohne Zögern Freunde/Angehörige bitten, wichtige Dinge für mich zu erledigen.	○	○	○	○	○
In meinem Umfeld gibt es Menschen, die Freude und Leid mit mir teilen.	○	○	○	○	○
Mit einigen Freunden/Angehörigen kann ich auch einmal ganz ausgelassen sein.	○	○	○	○	○

6. Haben Sie in den letzten 12 Monaten einen Verwandten, Freund/in, Nachbar/in (ca. wöchentlich) in seinem/ihrem oder Ihrem eigenen Zuhause gepflegt?

	O ja	O nein *->bitte weiter mit* ***Frage 37***

Es kommt vor, dass pflegende Angehörige mehrere Personen gleichzeitig unterstützen. Falls dies bei Ihnen der Fall ist, beantworten Sie bitte die nachfolgenden Fragen für die Person, die Sie am **meisten** unterstützen! Falls alle Personen denselben Umfang an Unterstützung erhalten, denken Sie bitte nur an eine Person.

**7. In welchem Verhältnis stehen Sie zu der Person, die Sie unterstützen? Sind Sie…**	O Ehe-/Lebenspartner/in	O (Schwieger-) Mutter/(Schwieger-) Vater
	O (Schwieger-/Paten-/Pflege-) Kind	O Sonstige/r Verwandte/r (z. B. Onkel/Tante, Schwester/Bruder, Enkelkind)
	O Freund/in	O Nachbar/in
	O Sonstiges	....

**8. Hat die Person, die Sie unterstützen, einen Pflegegrad?**	O ja	O nein *->bitte weiter mit* ***Frage 10***
	O Ich weiß es nicht *->bitte weiter mit* ***Frage 10***	

**9. Welchen Pflegegrad hat die Person, die Sie unterstützen?**	O Pflegegrad 1	O Pflegegrad 2
	O Pflegegrad 3	O Pflegegrad 4
	O Pflegegrad 5	O Ich weiß es nicht

MUSTER
Nicht ausfüllen!

10. Welche der folgenden Leistungen der Pflegekassen kennen Sie und welche nutzen Sie (bzw. der zu Pflegende), falls Sie jemanden pflegen?

**Pflegegeld**<br>Das Pflegegeld wird dem Betroffenen von der Pflegekasse überwiesen. Dieser kann über die Verwendung des Pflegegeldes frei verfügen und kann es an die betreuenden Personen weitergeben. (Mehrfachantworten möglich)	☐ Kenne ich, nutzen wir aber nicht<br>☐ Kenne ich und weiß nicht, ob es genutzt wird	☐ Kenne ich und nutzen wir<br>☐ unbekannt (und nutzen wir nicht)
**Pflegesachleistung**<br>Die Pflegesachleistungen umfassen häusliche Pflege, die von professionellen Pflegekräften in Form von Grundpflege und hauswirtschaftlicher Versorgung erbracht werden. (Mehrfachantworten möglich)	☐ Kenne ich, nutzen wir aber nicht<br>☐ Kenne ich und weiß nicht, ob es genutzt wird	☐ Kenne ich und nutzen wir<br>☐ unbekannt (und nutzen wir nicht)
**Teilstationäre Pflege**<br>Teilstationäre Pflege umfasst Leistungen der Tages- oder Nachtpflege. Sie kann die häusliche Pflege ergänzen, wenn diese nicht in ausreichendem Umfang möglich ist. Einen Anspruch auf teilstationäre Leistungen haben Pflegebedürftige der Pflegegrade 2 bis 5. (Mehrfachantworten möglich)	☐ Kenne ich, nutzen wir aber nicht<br>☐ Kenne ich und weiß nicht, ob es genutzt wird	☐ Kenne ich und nutzen wir<br>☐ unbekannt (und nutzen wir nicht)
**Vollstationäre Pflegeleistungen**<br>Die Pflegeversicherung zahlt bei vollstationärer Pflege pauschale Leistungen für pflegebedingte Aufwendungen einschließlich der Aufwendungen für Betreuung und die Aufwendungen für Leistungen der medizinischen Behandlungspflege in Pflegeheimen. (Mehrfachantworten möglich)	☐ Kenne ich, nutzen wir aber nicht<br>☐ Kenne ich und weiß nicht, ob es genutzt wird	☐ Kenne ich und nutzen wir<br>☐ unbekannt (und nutzen wir nicht)

**Entlastungsbetrag**<br>Der Entlastungsbetrag ergänzt die ambulanten und teilstationären Pflegeleistungen in der häuslichen Umgebung. **Alle** Pflegebedürftigen mit den **Pflegegraden 1 bis 5**, bei denen im häuslichen Bereich gepflegt wird, haben einen Anspruch auf den Entlastungsbetrag.<br>(Mehrfachantworten möglich)	☐ Kenne ich, nutzen wir aber nicht<br>☐ Kenne ich und nutzen wir<br>☐ Kenne ich und weiß nicht, ob es genutzt wird<br>☐ unbekannt (und nutzen wir nicht)
**Kurzzeitpflege**<br>Manche Pflegebedürftige sind für eine begrenzte Zeit auf vollstationäre Pflege angewiesen, zum Beispiel weil eine Krisensituation bei der häuslichen Pflege bewältigt oder der Übergang im Anschluss an einen Krankenhausaufenthalt geregelt werden muss. Für sie gibt es die Kurzzeitpflege in entsprechenden vollstationären Einrichtungen.<br>(Mehrfachantworten möglich)	☐ Kenne ich, nutzen wir aber nicht<br>☐ Kenne ich und nutzen wir<br>☐ Kenne ich und weiß nicht, ob es genutzt wird<br>☐ unbekannt (und nutzen wir nicht)
**Verhinderungspflege**<br>Macht die private Pflegeperson Urlaub oder ist sie vorübergehend an der Pflege gehindert, übernimmt die Pflegeversicherung die Kosten einer Ersatzpflege für maximal sechs Wochen je Kalenderjahr (Ab Pflegegrad 2).<br>(Mehrfachantworten möglich)	☐ Kenne ich, nutzen wir aber nicht<br>☐ Kenne ich und nutzen wir<br>☐ Kenne ich und weiß nicht, ob es genutzt wird<br>☐ unbekannt (und nutzen wir nicht)
**Pflegehilfsmittel**<br>Pflegehilfsmittel sind Geräte und Sachmittel, die zur Erleichterung der häuslichen Pflege oder zur Linderung der Beschwerden der Pflegebedürftigen beitragen oder den Pflegebedürftigen eine selbstständigere Lebensführung ermöglichen.<br>(Mehrfachantworten möglich)	☐ Kenne ich, nutzen wir aber nicht<br>☐ Kenne ich und nutzen wir<br>☐ Kenne ich und weiß nicht, ob es genutzt wird<br>☐ unbekannt (und nutzen wir nicht)
**Wohnumfeldverbesserung**<br>Die Pflegekasse kann für Pflegebedürftige der Pflegegrade 1 bis 5 auf Antrag bis zu 4.000 Euro als Zuschuss für Anpassungsmaßnahmen zahlen, die die häusliche Pflege in der Wohnung verbessern und die Pflegeperson(en) entlasten sollen.<br>(Mehrfachantworten möglich)	☐ Kenne ich, nutzen wir aber nicht<br>☐ Kenne ich und nutzen wir<br>☐ Kenne ich und weiß nicht, ob es genutzt wird<br>☐ unbekannt (und nutzen wir nicht)
**Wohngruppenzuschlag**<br>Mit dem Wohngruppenzuschlag werden Bewohnerinnen und Bewohner ambulant betreuter Wohngruppen unterstützt.<br>(Mehrfachantworten möglich)	☐ Kenne ich, nutzen wir aber nicht<br>☐ Kenne ich und nutzen wir<br>☐ Kenne ich und weiß nicht, ob es genutzt wird<br>☐ unbekannt (und nutzen wir nicht)

MUSTER
Nicht ausfüllen!

**11. In welchen Bereichen liegen Einschränkungen bei der Person, die Sie unterstützen, vor?**	○ körperlich<br>○ kognitiv/psychisch<br>○ beides<br>○ weiß nicht

**12. Wie lange helfen Sie der von Ihnen unterstützten Person bereits?**	○ unter 1 Jahr<br>○ 1 bis unter 3 Jahre<br>○ 3 bis unter 6 Jahre<br>○ 6 bis unter 9 Jahre<br>○ 9 Jahre und mehr

**13. Wie lange liegt bei der von Ihnen unterstützten Person eine Pflegestufe bzw. Pflegegrad vor?**<br>*Wenn Sie es nicht mehr genau wissen, dann schätzen Sie bitte.*	○ unter 1 Jahr<br>○ 1 bis unter 3 Jahre<br>○ 3 bis unter 6 Jahre<br>○ 6 bis unter 9 Jahre<br>○ 9 Jahre und mehr<br>○ Es liegt kein Pflegegrad vor.<br>○ Ich weiß es nicht

**14. Wie viele Stunden wenden Sie pro Woche auf, um der von Ihnen unterstützten Person zu helfen?** *Wenn der zeitliche Aufwand zwischen den Wochen variiert oder nicht genau bekannt ist, schätzen Sie bitte den durchschnittlichen wöchentlichen Aufwand.*	○ unter 5 Stunden<br>○ 5 bis unter 10 Stunden<br>○ 10 bis unter 20 Stunden<br>○ 20 bis unter 30 Stunden<br>○ 30 bis unter 40 Stunden<br>○ 40 Stunden und mehr

**15. Wie viele Privatpersonen aus der Familie, Nachbarschaft oder Bekanntschaft sind insgesamt an der Unterstützung und/oder Pflege beteiligt?**	O 1 ->*bitte weiter mit* ***Frage 17*** O 2<br>O 3 O 4<br>O mehr und zwar..

**16. Sind Sie die private Hauptpflegeperson, d.h. mehr als die anderen mit der Unterstützung und/oder Pflege befasst?**	O ja O nein<br>O Sonstiges, und zwar…
**17. Wohnen Sie und die Person, die Sie unterstützen, im selben Haushalt?**	O ja O nein

18. Inwieweit benötigt die von Ihnen unterstützte Person in den folgenden Bereichen Hilfe?

	gar nicht						sehr intensiv	Will keine Hilfe
**Körperpflege, Ernährung und Mobilität** (z. B. beim Duschen helfen, Essen anreichen, beim Umbetten, Lagern oder Transfer helfen, beim Toilettengang helfen)	O	O	O	O	O	O	O	O
**Medizinische/pflegerische Versorgung** (z. B. Blutzucker messen, Wunde versorgen, Kompressionsstrümpfe anziehen, mit Medikamenten versorgen)	O	O	O	O	O	O	O	O
**Führung des Haushalts** (z. B. putzen, kochen, waschen, einkaufen)	O	O	O	O	O	O	O	O
**Organisation, Koordination und Verwaltung von Hilfe und Pflege** (z. B. Leistungsanträge stellen, Pflegedienst koordinieren, Behördengänge, Arztbesuche)	O	O	O	O	O	O	O	O
**Betreuung und Beschäftigung im Alltag für die pflegebedürftige Person** innerhalb und außerhalb des Haushalts (z. B. Gesellschaft leisten, gemeinsame Aktivitäten)	O	O	O	O	O	O	O	O

MUSTER
Nicht ausfüllen!

19. Wie intensiv helfen Sie der von Ihnen unterstützten Person in den folgenden Bereichen?

	gar nicht						sehr intensiv	Braucht keine Hilfe
**Körperpflege, Ernährung und Mobilität** (z. B. beim Duschen helfen, Essen anreichen, beim Umbetten, Lagern oder Transfer helfen, beim Toilettengang helfen)	O	O	O	O	O	O	O	O
**Medizinische/pflegerische Versorgung** (z. B. Blutzucker messen, Wunde versorgen, Kompressionsstrümpfe anziehen, mit Medikamenten versorgen)	O	O	O	O	O	O	O	O
**Führung des Haushalts** (z. B. putzen, kochen, waschen, einkaufen)	O	O	O	O	O	O	O	O
**Organisation, Koordination und Verwaltung von Hilfe und Pflege** (z. B. Leistungsanträge stellen, Pflegedienst koordinieren, Behördengänge, Arztbesuche)	O	O	O	O	O	O	O	O
**Betreuung und Beschäftigung im Alltag für die pflegebedürftige Person** innerhalb und außerhalb des Haushalts (z. B. Gesellschaft leisten, gemeinsame Aktivitäten)	O	O	O	O	O	O	O	O

20. Wird die von Ihnen unterstützte Person zusätzlich durch einen Pflegedienst betreut?

O ja
O nein ->*bitte weiter mit* ***Frage 22***

21. Wie intensiv hilft der Pflegedienst der von Ihnen unterstützten Person in den folgenden Bereichen?

	gar nicht						sehr intensiv	Braucht keine Hilfe
**Körperpflege, Ernährung und Mobilität** (z. B. beim Duschen helfen, Essen anreichen, beim Umbetten, Lagern oder Transfer helfen, beim Toilettengang helfen)	O	O	O	O	O	O	O	O
**Medizinische/pflegerische Versorgung** (z. B. Blutzucker messen, Wunde versorgen, Kompressionsstrümpfe anziehen, mit Medikamenten versorgen)	O	O	O	O	O	O	O	O
**Führung des Haushalts** (z. B. putzen, kochen, waschen, einkaufen)	O	O	O	O	O	O	O	O
**Organisation, Koordination und Verwaltung von Hilfe und Pflege** (z. B. Leistungsanträge stellen, Pflegedienst koordinieren, Behördengänge, Arztbesuche)	O	O	O	O	O	O	O	O
**Betreuung und Beschäftigung im Alltag für die pflegebedürftige Person** innerhalb und außerhalb des Haushalts (z. B. Gesellschaft leisten, gemeinsame Aktivitäten)	O	O	O	O	O	O	O	O

22. Haben Sie schon eine Pflegeberatung in Anspruch genommen?

- O ja, einmal
- O ja, mehrmals
- O ja, regelmäßig
- O nein
- O eine andere, ebenfalls mit der Pflege betraute, Person hat eine Beratung in Anspruch genommen.
- O weiß nicht

MUSTER
Nicht ausfüllen!

23. Wie lange ist es her, dass Sie bzw. die von Ihnen unterstützte Person eine (Pflege-) Beratung hatten? *Wenn Sie es nicht mehr genau wissen, dann schätzen Sie bitte.*

- O bis zu 3 Monate
- O mehr als 3 bis 6 Monate
- O mehr als 6 bis 9 Monate
- O mehr als 9 bis 12 Monate
- O mehr als 12 Monate

24. Wie fand die letzte Pflegeberatung statt?

- O persönlich, mündlich
- O per Telefon ->*bitte weiter mit* ***Frage 26***
- O per E-Mail ->*bitte weiter mit* ***Frage 26***

25. Wo fand die letzte Pflegeberatung statt?

- O zu Hause
- O nicht zu Hause, aber im Wohnort
- O nicht im Wohnort, aber im Landkreis
- O in einem anderen Landkreis in Sachsen
- O in Sachsen-Anhalt
- O in Berlin
- O sonstiges, und zwar… ....

-> Bitte weiter mit ***Frage 27***

26. Wo befand sich die Pflegeberatung, mit der Sie telefoniert bzw. per E-Mail geschrieben haben?

- O im Wohnort
- O nicht im Wohnort, aber im Landkreis
- O in einem anderen Landkreis in Sachsen
- O in Sachsen-Anhalt
- O in Berlin
- O sonstiges, und zwar…

-> Wenn nicht Sachsen angegeben wurde, haben Sie zum Zeitpunkt der Beratung in diesem Bundesland gelebt?

O ja O nein

27. Inwieweit kennen Sie folgende Angebote? Benötigen Sie solche Angebote und sind Sie schon ein oder mehrmals darin beraten worden? [Mehrfachantworten möglich]

Pflegekurse	○ Ja, kenne ich<br>○ Ich bin darin schon beraten worden	○ Ja, benötige ich
Freistellungsmöglichkeiten nach dem Pflegezeit- und Familienpflegezeitgesetz	○ Ja, kenne ich<br>○ Ich bin darin schon beraten worden	○ Ja, benötige ich
Pflegeunterstützungsgeld	○ Ja, kenne ich<br>○ Ich bin darin schon beraten worden	○ Ja, benötige ich
Entlastungsbetrag	○ Ja, kenne ich<br>○ Ich bin darin schon beraten worden	○ Ja, benötige ich
Pflegesach- und Kombinationsleistungen	○ Ja, kenne ich<br>○ Ich bin darin schon beraten worden	○ Ja, benötige ich
Angebote zur Unterstützung im Alltag	○ Ja, kenne ich<br>○ Ich bin darin schon beraten worden	○ Ja, benötige ich
Angebote zur Verhinderungspflege	○ Ja, kenne ich<br>○ Ich bin darin schon beraten worden	○ Ja, benötige ich
Tages- und Nachtpflege	○ Ja, kenne ich<br>○ Ich bin darin schon beraten worden	○ Ja, benötige ich
Angebote der Kurzzeitpflege	○ Ja, kenne ich<br>○ Ich bin darin schon beraten worden	○ Ja, benötige ich
Präventions- und Rehabilitationsmaßnahmen	○ Ja, kenne ich<br>○ Ich bin darin schon beraten worden	○ Ja, benötige ich
Angebote der Selbsthilfe, z. B. Angehörigengruppen	○ Ja, kenne ich<br>○ Ich bin darin schon beraten worden	○ Ja, benötige ich
Angebote von Ärzten/Psychotherapeuten	○ Ja, kenne ich<br>○ Ich bin darin schon beraten worden	○ Ja, benötige ich
Hilfs-/Pflegehilfsmittel(-Beratung) und technische Hilfen	○ Ja, kenne ich<br>○ Ich bin darin schon beraten worden	○ Ja, benötige ich
Anpassung des Wohnumfeldes	○ Ja, kenne ich<br>○ Ich bin darin schon beraten worden	○ Ja, benötige ich
Sonstiges, und zwar…..		

MUSTER
Nicht ausfüllen!

28. Wie hilfreich empfanden Sie die Angebote?

Bewerten Sie bitte auf einer Skala von „gar nicht hilfreich“ bis „sehr hilfreich“

	gar nicht hilfreich				sehr hilfreich
Pflegekurse	○	○	○	○	○
Freistellungsmöglichkeiten nach dem Pflegezeit- und Familienpflegezeitgesetz	○	○	○	○	○
Pflegeunterstützungsgeld	○	○	○	○	○
Entlastungsbetrag	○	○	○	○	○
Pflegesach- und Kombinationsleistungen	○	○	○	○	○
Angebote zur Unterstützung im Alltag	○	○	○	○	○
Angebote zur Verhinderungspflege	○	○	○	○	○
Tages- und Nachtpflege	○	○	○	○	○
Angebote der Kurzzeitpflege	○	○	○	○	○
Präventions- und Rehabilitationsmaßnahmen	○	○	○	○	○
Angebote der Selbsthilfe, z. B. Angehörigengruppen	○	○	○	○	○
Angebote von Ärzten/Psychotherapeuten	○	○	○	○	○
Hilfs-/Pflegehilfsmittel(-Beratung) und technische Hilfen	○	○	○	○	○
Anpassung des Wohnumfeldes	○	○	○	○	○
(Ihre Antwort aus Frage 27)	○	○	○	○	○

MUSTER
Nicht ausfüllen!

**29. Wie zufrieden waren Sie mit der Beratung?** *Falls es Mehrere waren, dann denken Sie bitte an die letzte Beratung.*	○ sehr unzufrieden<br>○ eher unzufrieden<br>○ nicht wirklich zufrieden<br>○ einigermaßen zufrieden<br>○ sehr zufrieden

**30. Wie lange mussten Sie auf einen Termin warten?** (Falls Sie persönliche Beratung angekreuzt hatten.)	○ bis zu einem Tag<br>○ bis zu einer Woche<br>○ bis zu einem Monat<br>○ bis zu drei Monaten<br>○ bis zu einem halben Jahr<br>○ länger als ein halbes Jahr<br>○ Keine persönliche Beratung

**31. Wie schnell wurde Ihre E-Mail beantwortet?** (Falls Sie Beratung per E-Mail angekreuzt hatte)	○ innerhalb von einem Tag<br>○ innerhalb von einer Woche<br>○ Ich habe länger als eine Woche auf eine Antwort gewartet.<br>○ Keine Beratung per E-Mail

**32. Reduzieren Sie aufgrund der Pflege Ihre Arbeitszeit?**	○ Ja<br>○ Nein<br>○ Ich wusste nicht, dass es diese Möglichkeit gibt.

**33. Zahlt die Pflegekasse für Sie als pflegende/r Angehörige/r Rentenversicherungsbeiträge?**	○ ja<br>○ nein<br>○ Ich weiß es nicht

**34. In den Medien wurde hin und wieder darüber berichtet, dass in der Pflege mehr Leistungen abgerechnet als tatsächlich erbracht werden. Haben Sie bei der häuslichen Pflege auch schon einmal erlebt, dass Leistungen bei Ihnen [bei der Person, die Sie unterstützen] abgerechnet wurden, die Sie [sie] eigentlich nicht bekommen haben/hat?**	O nie O einmal O mehrmals O Ich weiß es nicht.

**35. Befanden Sie sich schon einmal in der Situation, dass Sie die Unterstützung der pflegebedürftigen Person vollständig oder teilweise abgegeben haben?**	O ja, vollständig O ja, teilweise O nein

36. Warum wollten/mussten Sie sie abgeben?

- O eigene körperliche Überforderung
- O eigene seelische Überforderung
- O selbst krank
- O keine Erfahrung
- O Inkontinenz beim Pflegebedürftigen
- O Demenz beim Pflegebedürftigen
- O Andere Gründe, und zwar…

->Bitte weiter mit ***Frage 47***

MUSTER
Nicht ausfüllen!

Fragebogenabschnitt: Für nicht pflegende Angehörige

37. Inwieweit kommen Sie mit dem Thema Pflege von Angehörigen in Berührung? Mehrfachantworten möglich

- O Ich habe mich noch nie mit dem Thema Pflege beschäftigt.
- O Ich habe mich mit dem Thema Pflege bereits beschäftigt.
- O Ich habe eine(n) Verwandten, Freund oder Nachbarn gepflegt.
- O Ich habe selbst einen Pflegegrad.
- O In meinem Haushalt leben eine oder mehrere Personen mit einem Pflegegrad.
- O Eine oder mehrere Personen in meiner Verwandtschaft haben einen Pflegegrad.
- O Eine oder mehrere Personen in meinem Freundeskreis haben einen Pflegegrad.
- O Keiner in meiner Verwandtschaft oder in meinem Freundeskreis besitzt einen Pflegegrad.
- O Sonstiges, und zwar….

38. Falls Sie in die Situation kommen, dass ein naher Angehöriger Pflege benötigt und sie um Hilfe gebeten werden. Was würden Sie tun? Stichpunkte genügen

39. Wissen Sie, wo man…

… sich beraten lassen kann?	O Ja	O Nein
… Leistungen beantragen kann?	O Ja	O Nein
… einen Antrag stellen kann?	O Ja	O Nein

Bei den folgenden Fragen interessiert uns, welche Kenntnisse Sie persönlich von den Leistungen der Pflegekasse haben. Bitte antworten Sie spontan und nutzen Sie möglichst keine Hilfsmittel. Sie helfen uns damit, den Wissensstand der Bevölkerung besser einzuschätzen.

40. Kennen bzw. wissen Sie, …

…wo die nächste Pflegeberatung ist?	O ja O nein<br>O Wo?
…wo sich der nächste Pflegestützpunkt befindet?	O ja O nein<br>O Wo?
…dass die Beratung auch nach Hause kommen kann?	O ja O nein
…dass Sie Ihre Arbeitszeit für die Pflege von Angehörigen reduzieren können?	O ja O nein
…dass für pflegende Angehörige Rentenversicherungsbeiträge gezahlt werden?	O ja O nein

41. Welche der folgenden Leistungen der Pflegekassen kennen Sie?

MUSTER
Nicht ausfüllen!

Pflegegeld	O Kenne ich<br>O Habe ich bzw. wir schon einmal genutzt	O Unbekannt
Pflegesachleistungen	O Kenne ich<br>O Habe ich bzw. wir schon einmal genutzt	O Unbekannt
Teilstationäre Pflege	O Kenne ich<br>O Habe ich bzw. wir schon einmal genutzt	O Unbekannt
Vollstationäre Pflegeleistungen	O Kenne ich<br>O Habe ich bzw. wir schon einmal genutzt	O Unbekannt
Entlastungsbetrag	O Kenne ich<br>O Habe ich bzw. wir schon einmal genutzt	O Unbekannt
Pflegehilfsmittel	O Kenne ich<br>O Habe ich bzw. wir schon einmal genutzt	O Unbekannt
Wohnumfeldverbesserung	O Kenne ich<br>O Habe ich bzw. wir schon einmal genutzt	O Unbekannt
Wohngruppenzuschlag	O Kenne ich<br>O Habe ich bzw. wir schon einmal genutzt	O Unbekannt
Ausgleichzahlungen von Rentenversicherungsbeiträgen der Pflegekasse für pflegende/r Angehörige/r	O Kenne ich<br>O Habe ich bzw. wir schon einmal genutzt	O Unbekannt
Fristen bei der Antragstellung eines Erstantrages zum Pflegegrad	O Kenne ich<br>O Habe ich bzw. wir schon einmal genutzt	O Unbekannt

**42. Haben Sie schon einmal die Pflege bzw. die Unterstützung einer pflegebedürftigen Person vollständig oder teilweise abgegeben?**	○ ja, vollständig<br>○ ja, teilweise<br>○ nein- Bitte weiter mit Frage 45
**43. Warum haben Sie die Unterstützung vollständig oder teilweise abgegeben?**<br>(Mehrfachantworten möglich)	☐ eigene körperliche Überforderung<br>☐ eigene seelische Überforderung<br>☐ selbst krank<br>☐ keine Erfahrung<br>☐ Inkontinenz beim Pflegebedürftigen<br>☐ Demenz beim Pflegebedürftigen<br>☐ Andere Gründe, und zwar…
**44. Welches Verhältnis hatten Sie zu der pflegebedürftigen Person?**	○ Ehe-/Lebenspartner/in<br>○ (Schwieger-) Mutter/(Schwieger-) Vater<br>○ (Schwieger-/Paten-/Pflege-) Kind<br>○ Sonstige/r Verwandte/r (z. B. Onkel/Tante, Schwester/Bruder, Enkelkind)<br>○ Freund/in<br>○ Nachbar/in<br>○ Sonstige, und zwar
*-> Bitte weiter mit **Frage 47***	

**45. Wen könnten Sie sich vorstellen zu pflegen bzw. unterstützen?**<br>(Mehrfachantworten möglich)	☐ Ehe-/Lebenspartner/in<br>☐ (Schwieger-) Mutter/(Schwieger-) Vater<br>☐ (Schwieger-/Paten-/Pflege-) Kind<br>☐ Sonstige/r Verwandte/r (z. B. Onkel/Tante, Schwester/Bruder, Enkelkind)<br>☐ Freund/in<br>☐ Nachbar/in<br>☐ Sonstige, und zwar…

MUSTER
Nicht ausfüllen!

46. Wie wahrscheinlich ist es, dass Sie folgende Bereiche der Pflege übernehmen würden?

	Sehr unwahrscheinlich				Sehr wahrscheinlich
**Körperpflege, Ernährung und Mobilität** (z. B. beim Duschen helfen, Essen anreichen, beim Umbetten, Lagern oder Transfer helfen, beim Toilettengang helfen)	○	○	○	○	○

	gar nicht						sehr intensiv	Braucht keine Hilfe
**Medizinische/pflegerische Versorgung** (z. B. Blutzucker messen, Wunde versorgen, Kompressionsstrümpfe anziehen, mit Medikamenten versorgen)	○	○	○	○	○	○	○	○
**Führung des Haushalts** (z. B. putzen, kochen, waschen, einkaufen)	○	○	○	○	○	○	○	○
**Organisation, Koordination und Verwaltung von Hilfe und Pflege** (z. B. Leistungsanträge stellen, Pflegedienst koordinieren, Behördengänge, Arztbesuche)	○	○	○	○	○	○	○	○
**Betreuung und Beschäftigung im Alltag für die pflegebedürftige Person** innerhalb und außerhalb des Haushalts (z. B. Gesellschaft leisten, gemeinsame Aktivitäten)	○	○	○	○	○	○	○	○

Diese Fragen sind wieder für alle Teilnehmer/innen.

47. Nun folgen noch einige Aussagen zum Thema Pflege.
Bitte kreuzen Sie an, inwieweit Sie der Aussage zustimmen:

	stimme gar nicht zu	stimme eher nicht zu	unentschieden	stimme eher zu	stimme voll zu
Wenn ich die Wahl habe, möchte ich lieber von einer einheimischen als von einer ausländischen Pflegekraft versorgt werden.	○	○	○	○	○
Wenn in meiner Verwandtschaft ein Pflegefall eintritt, weiß ich nicht, was zu tun ist.	○	○	○	○	○
Für die Betreuung von Angehörigen müsste man von der Arbeitsstelle freigestellt werden.	○	○	○	○	○
Ich habe Angst, dass meine Kinder für meine Pflege zuzahlen müssen.	○	○	○	○	○
Viele ambulante Pflegedienste rechnen mehr Leistungen ab, als erbracht werden.	○	○	○	○	○
Jede/r sollte eine private Zusatzversicherung für Pflegerisiken abschließen.	○	○	○	○	○

48. Wie schätzen Sie die Versorgung von pflegebedürftigen Menschen in folgenden Bereichen ein?

	sehr schlecht										sehr gut
zu Hause durch Angehörige	○	○	○	○	○	○	○	○	○	○	○
zu Hause durch Pflegedienst	○	○	○	○	○	○	○	○	○	○	○
in Pflegeheimen	○	○	○	○	○	○	○	○	○	○	○

49. Wie würden Sie Ihre derzeitige finanzielle Situation beschreiben?

○ Es reicht vorne und hinten nicht.
○ Ich komme gerade so über die Runden.
○ Im Großen und Ganzen komme ich zurecht.
○ Ich bin gut versorgt und kann mir Einiges leisten.
○ Ich muss mich in keiner Weise einschränken.

MUSTER
Nicht ausfüllen!

50. Die folgenden Aussagen betreffen Ihr Wohlbefinden in den letzten vier Wochen.
Kreuzen Sie zu jeder Aussage an, was für Sie am ehesten zutrifft. Beantworten Sie bitte jede Frage!

	stimmt nicht	stimmt wenig	stimmt überwiegend	stimmt genau
Mir bleibt genügend Zeit für meine eigenen Interessen und Bedürfnisse.	○	○	○	○
Ich fühle mich oft körperlich erschöpft.	○	○	○	○
Ich habe hin und wieder den Wunsch, aus meiner Situation „auszubrechen“.	○	○	○	○
Ich kann mich von Herzen freuen.	○	○	○	○
Mein Lebensstandard hat sich in den letzten Jahren verringert.	○	○	○	○
Ich fühle mich von Menschen, die ich unterstütze, manchmal ausgenutzt.	○	○	○	○
Meine derzeitigen Aufgaben kosten viel von meiner eigenen Kraft.	○	○	○	○
Ich fühle mich “hin und her gerissen” zwischen den unterschiedlichen Anforderungen meiner Umgebung (z.B. Arbeit, Familie, Pflege).	○	○	○	○
Ich habe das Gefühl, alles “im Griff” zu haben.	○	○	○	○
Aufgrund meiner derzeitigen Aufgaben leidet meine Beziehung zu Familienangehörigen, Verwandten, Freunden und Bekannten.	○	○	○	○
Das Schicksal von kranken Menschen in meiner Umgebung macht mich traurig.	○	○	○	○
Durch meine Leistungen bekomme ich Anerkennung/Dankbarkeit.	○	○	○	○

Im Folgenden stellen wir Ihnen noch einige allgemeine Fragen zu Ihrer Person und zu der Person, die Sie unterstützen. Durch die Beantwortung dieser Fragen helfen Sie, die Unterstützungsleistungen auf die individuellen Anforderungen und Wünsche pflegender Angehöriger anzupassen.

**51. Ihr Geschlecht?**	○ weiblich ○ männlich ○ divers
**52. In welchem Jahr sind Sie geboren?**	☐☐

**53. Sind Sie in Deutschland geboren?**	○ ja ○ nein, und zwar ☐
**54. In welcher Gemeinde/Stadt leben Sie?** *Bitte geben Sie die Postleitzahl an!*	☐☐☐☐☐
**55. Welchen Familienstand haben Sie?**	○ ledig ○ verheiratet / in Partnerschaft lebend ○ geschieden/getrennt ○ verwitwet

**56. Haben Sie Kinder?**	○ Ja ○ Nein *->Bitte weiter mit* ***Frage 59***
**57. Wie viele Kinder haben Sie?**	☐☐
**58. Lebt noch mindestens ein Kind unter 18 Jahre in Ihrem Haushalt?**	○ Ja ○ Nein
**59. Wie viele Personen leben ständig in Ihrem Haushalt, Sie selbst eingeschlossen?** Denken Sie dabei bitte auch an alle im Haushalt lebenden Kinder.	☐☐

60. Welchen höchsten Bildungsabschluss haben Sie?

- ○ (zurzeit noch) ohne Abschluss
- ○ Volks-/ Hauptschulabschluss / POS 8. oder 9. Klasse
- ○ Mittlere Reife / Realschulabschluss / POS 10. Klasse
- ○ Fachhochschulreife
- ○ Abitur / EOS
- ○ Fachschulabschluss
- ○ Hochschulabschluss
- ○ Anderer Abschluss, und zwar: ☐

MUSTER
Nicht ausfüllen!

61. Sind Sie zurzeit erwerbstätig?

erwerbstätig/selbstständig

- ○ in Vollzeit
- ○ in Teilzeit (32h bis unter 38,5 h wöchentlich)
- ○ in Teilzeit (21h bis unter 32h wöchentlich)
- ○ in Teilzeit (weniger als 21h wöchentlich)
- ○ in Elternzeit
- ○ arbeitslos/-suchend

**nicht erwerbstätig**
O (zurzeit noch) ohne Abschluss<br>O in Aus- bzw. Weiterbildung (zum Beispiel: Studium, Berufsausbildung etc.)<br>O Hausfrau/-mann<br>O Rentner/-in, Pensionär/-in, im Vorruhestand<br>O dauerhaft erwerbsunfähig<br>O Bundesfreiwilligendienst

**62. Sind oder waren Sie in der Gesundheitsbranche tätig?**	O Nein<br>O Ja, und zwar…..

63. Erhalten Sie oder ein anderes Haushaltsmitglied derzeit eine der folgenden staatlichen Leistungen?

Arbeitslosengeld II (Hartz IV)	O Ja, ich<br>O Ja, ich und ein anderes Haushaltsmitglied	O Ja, ein anderes Haushaltsmitglied<br>O nein
Grundsicherung im Alter und bei Erwerbsminderung (Sozialhilfe)	O Ja, ich<br>O Ja, ich und ein anderes Haushaltsmitglied	O Ja, ein anderes Haushaltsmitglied<br>O nein
Hilfe zum Lebensunterhalt (Sozialhilfe)	O Ja, ich<br>O Ja, ich und ein anderes Haushaltsmitglied	O Ja, ein anderes Haushaltsmitglied<br>O nein
Hilfe zur Pflege (Sozialhilfe)	O Ja, ich<br>O Ja, ich und ein anderes Haushaltsmitglied	O Ja, ein anderes Haushaltsmitglied<br>O nein
Wohngeld (Miet- und Lastenzuschuss)	O Ja, ich<br>O Ja, ich und ein anderes Haushaltsmitglied	O Ja, ein anderes Haushaltsmitglied<br>O nein
Sonstige Sozialhilfeleistungen	O Ja, ich<br>O Ja, ich und ein anderes Haushaltsmitglied	O Ja, ein anderes Haushaltsmitglied<br>O nein

MUSTER
Nicht ausfüllen!

64. Wie hoch ist das monatliche Nettoeinkommen Ihres Haushaltes insgesamt?

Gemeint ist dabei die Summe, die sich aus Lohn, Gehalt, Einkommen aus selbstständiger Tätigkeit, Rente oder Pension ergibt. Rechnen Sie bitte auch die Einkünfte aus öffentlichen Beihilfen, Einkommen aus Vermietung, Verpachtung, Wohngeld, Kindergeld und sonstige Einkünfte hinzu. Ziehen Sie dann Steuern, Betriebsausgaben und Sozialversicherungsbeiträge ab.	O Unter 500 €<br>O 750 € bis unter 1.000 €<br>O 1.250 € bis unter 1.500 €<br>O 1.750 € bis unter 2.000 €<br>O 2.250 € bis unter 2.500 €<br>O 3.000 € bis unter 3.500 €<br>O 4.000 € bis unter 4.500 €<br>O 5.000 € bis unter 6.000 €<br>O Über 8.000 €	O 500 € bis unter 750 €<br>O 1.000 € bis unter 1.250 €<br>O 1.500 € bis unter 1.750 €<br>O 2.000 € bis unter 2.250 €<br>O 2.500 € bis unter 3.000 €<br>O 3.500 € bis unter 4.000 €<br>O 4.500 € bis unter 5.000 €<br>O 6.000 € bis unter 8.000 €<br>O Keine Angabe

->Falls Sie keine Angehörigen pflegen, bitte weiter mit ***Frage 68***

Zum Schluss stellen wir Ihnen noch einige allgemeine Fragen zu der Person, die Sie unterstützen.

**65. Welches Geschlecht hat die Person, die Sie unterstützen?**	O männlich O weiblich O divers
**66. In welchem Jahr ist die Person, die Sie unterstützen, geboren?**	
**67. Ist die Person, die Sie unterstützen, in Deutschland geboren?**	O ja O nein, sondern in

68. Möglicherweise haben wir den einen oder anderen für Sie wichtigen Punkt im Fragebogen nicht angesprochen. Sofern Sie noch Ergänzungen, Anmerkungen oder kritische Hinweise haben, bitten wir Sie, uns diese hier mitzuteilen:

MUSTER
Nicht ausfüllen!

Vielen Dank für Ihre Teilnahme!

Bitte legen Sie den ausgefüllten Fragebogen in den beiliegenden Rückumschlag. Der Rückumschlag ist bereits mit Adresse und Porto versehen (d.h. Sie müssen keine Briefmarke aufkleben). Den Fragebogen senden Sie bitte bis spätestens zum 2. Dezember an:

Prof. Dr. Tom Schaal, Professur für Management im Gesundheitswesen
Westsächsische Hochschule Zwickau, Fakultät Gesundheits- und Pflegewissenschaften
08012 Zwickau, Postfach 20 10 37
